# Supplementary material for: Dynamic subcellular proteomics identifies regulators of adipocyte insulin action
Source: Nat Commun. 2026 Feb 28;17:3310. doi: 10.1038/s41467-026-70116-9 (PMC13066455; doi:10.1038/s41467-026-70116-9)
Supplement: Supplementary file 1 — Supplementary Information [file 41467_2026_70116_MOESM1_ESM.pdf]

Supplementary Figure 1

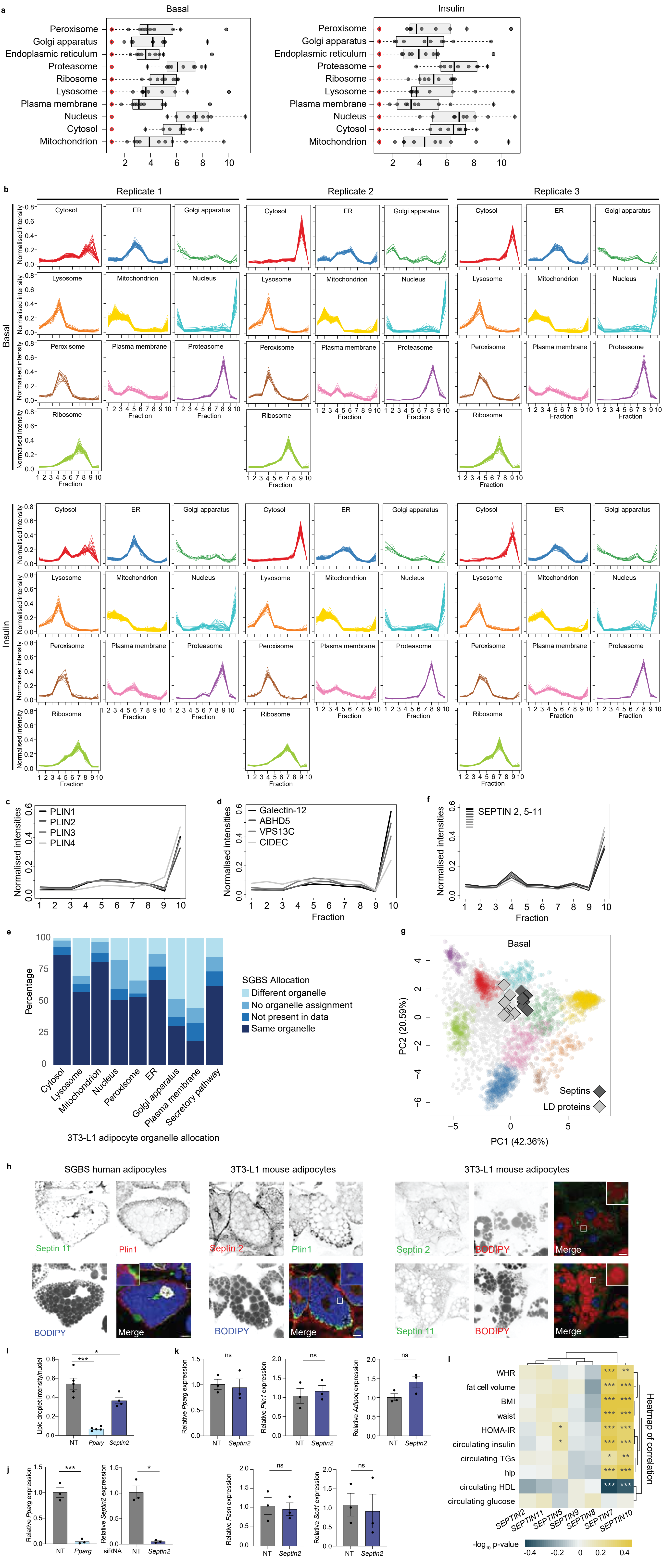

Supplementary Figure 1: a) Box plots representing normalised QSep scores between organelles for marker proteins in basal and insulin-stimulated adipocyte subcellular maps generated using LOPIT-DC. Each grey dot represents a QSep score between the organelle (Y-axis) and each other compartment. Higher QSep scores indicate more distinct resolution between compartments. Distances within a cluster are shown in red. b) Distribution of marker proteins (n = 531) across fractions in each replicate. Organelles coloured as red = cytosol, dark blue = endoplasmic reticulum, dark green = Golgi apparatus, orange = lysosome, yellow = mitochondrion, light blue = nucleus, brown = peroxisome, pink = plasma membrane, purple = proteasome, green = ribosome, grey = undefined. c-d) Mean distribution of (c) perilipin proteins, (d) other known lipid droplet associated proteins across fractions under basal conditions (n=3 biological replicates). e) Comparison of organelle allocation of proteins present in 3T3-L1 adipocytes to allocation in SGBS adipocytes[18]. f) Mean distribution of septin proteins identified (septin 2, 5-11) across fractions under basal conditions (n = 3 biological replicates). g) PCA projection of basal LOPIT-DC map with septins (2, 5-11, dark grey diamonds) and lipid droplet marker proteins (PLIN1-4, CIDEC, VPS13C, galectin-12, ABHD5, light grey diamonds) highlighted. h) Immunostaining of Septin 2, Septin 11, Plin1 and BODIPY^{TM} 493/503 in SGBS human adipocytes and 3T3-L1 mouse adipocytes (scale bar = 10 μm). i) Mean lipid droplet intensity in 3T3-L1 adipocytes following siRNA-mediated depletion of *Pparg* or *Septin2* on day 8 of differentiation, normalised to number of nuclei/well (n = 4-5 biological replicates). j) Relative expression of *Pparg and Septin2* in 3T3-L1 and adipocytes after siRNA-mediated knockdown during differentiation (n = 3 biological replicates). k) Relative expression of *Pparg, Plin1, Adipoq, Fasn and Scd1* normalised to *Actb* in 3T3-L1 adipocytes following *Septin2* siRNA-mediated depletion during adipogenesis (n = 3 biological replicates). l) Spearman's correlation of septin protein expression in omental adipose with metabolic clinical features. Figure generated from adiposetissue.org[26] using data from[27-47] (*** pFDR <0.001, ** pFDR < 0.01, * pFDR < 0.05). All data represented as mean ± SEM (i- k). n.s. non-significant; *$p < 0.05$; **$p < 0.01$; ***$p < 0.01$; by ordinary one-way ANOVA (i) or by paired two-tailed Student's *t*-test (j,k). Source data are provided as a Source Data File.

Supplementary Figure 2

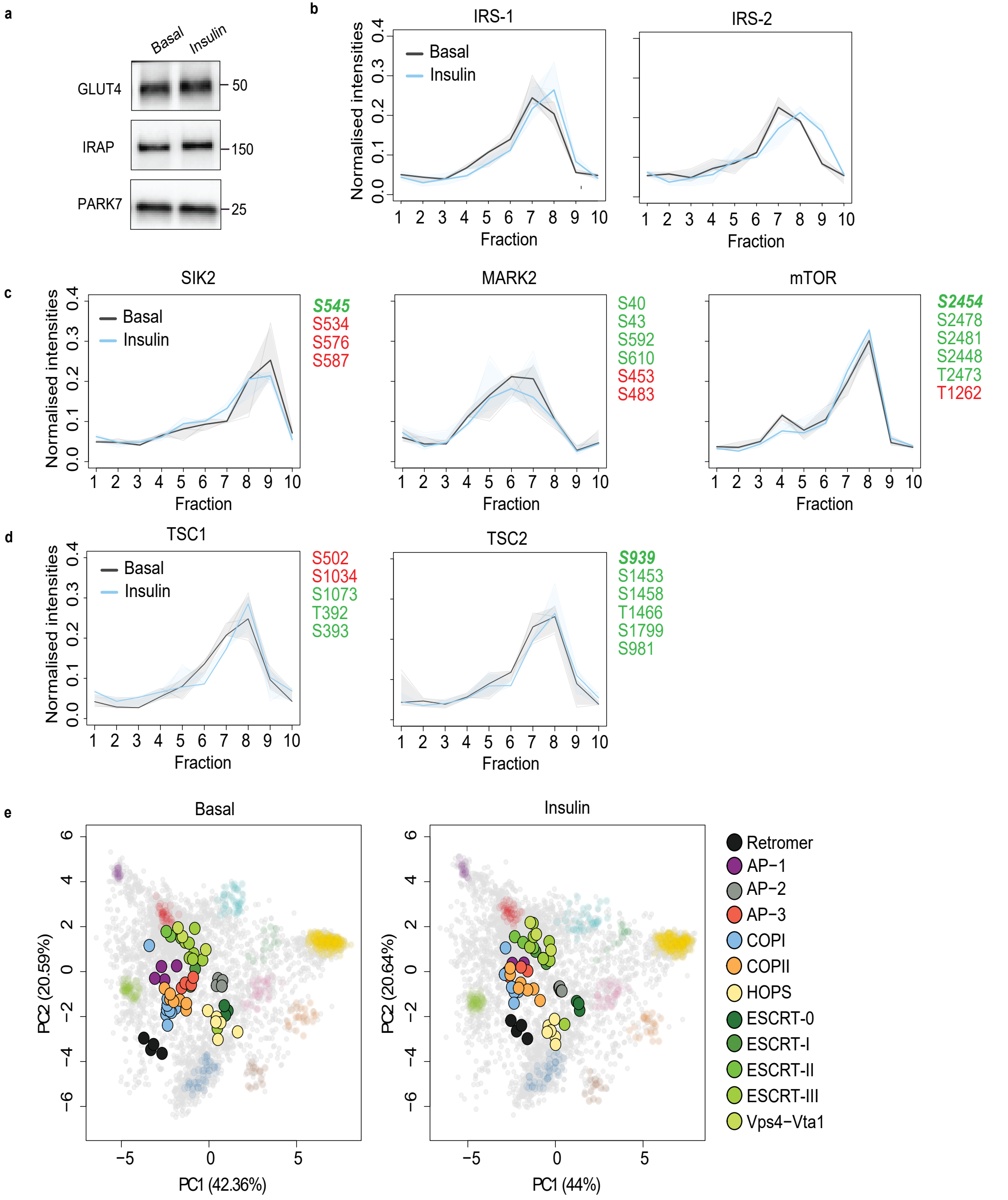

Supplementary Figure 2: a) Western blot analysis of IRAP and GLUT4 abundance in unstimulated (basal) or insulin-stimulated 3T3-L1 adipocytes (100 nM, 30 min). Representative blot shown, n = 4 biological replicates. b-d) Distribution of (b) IRS-1 and IRS-2, (c) SIK2, MARK2 and mTOR, and (d) TSC1/2 subunits across fractions in basal (grey) and insulin-treated (blue) adipocytes (solid line = mean, shaded area 95% CI, n = 3 per condition), and insulin-regulated phosphorylation sites reported in Fazakerley (2023)[10] and Humphrey (2013)[1] (green = increased and red = decreased phosphorylation, regular font = found in either Fazakerley and Humphrey, bold italic = found in both Fazakerley and Humphrey). IRS-1/2 have >20 regulated phosphosites and are listed in Supplementary Table 1. e) PCA projection of concatenated basal (left) and insulin (right) LOPIT-DC data with cytosolic cargo sorting complexes highlighted. Only subunits found in all 6 replicates are shown. Organelle marker proteins coloured as as red = cytosol, dark blue = endoplasmic reticulum, dark green = Golgi apparatus, orange = lysosome, yellow = mitochondrion, light blue = nucleus, brown = peroxisome, pink = plasma membrane, purple = proteasome, green = ribosome, grey = undefined. Cytosolic sorting complexes coloured as Retromer (black) - VPS26A, VPS26B, VPS29, VPS35; ESCRT-0 (dark green) - HRS, STAM2; ESCRT-I (medium-dark green) - VPS23, VPS37C; ESCRT-II (medium green) - SNF8, VPS25, VPS36; ESCRT-III (light green) - CHMP1A, CHMP2A, CHMP2B, CHMP3, CHMP4B, CHMP5, CHMP6, IST1; Vps4-Vta1 (yellow-green) - VPS4A, VPS4B, VTA1; AP-1 (purple) - AP1B1, AP1G1, AP1M1, AP1S1; AP-2 (grey) - AP2A1, AP2A2, AP2S1, AP2M1, AP2B1; AP-3 (red) - AP3B1, AP3D1, AP3M1, AP3S1; COPI (blue) - COPA, COPB1, COPB2, COPE, COPG1, COPG2, COPZ1, COPZ2, ARCN1; COPII (orange) - SAR1A, SAR1B, SEC13, SEC23A, SEC23B, SEC24A, SEC31A; HOPS (yellow) - VPS11, VPS16, VPS18, VPS33A, VPS39, VPS41. Source data are provided as a Source Data File.

Supplementary Figure 3

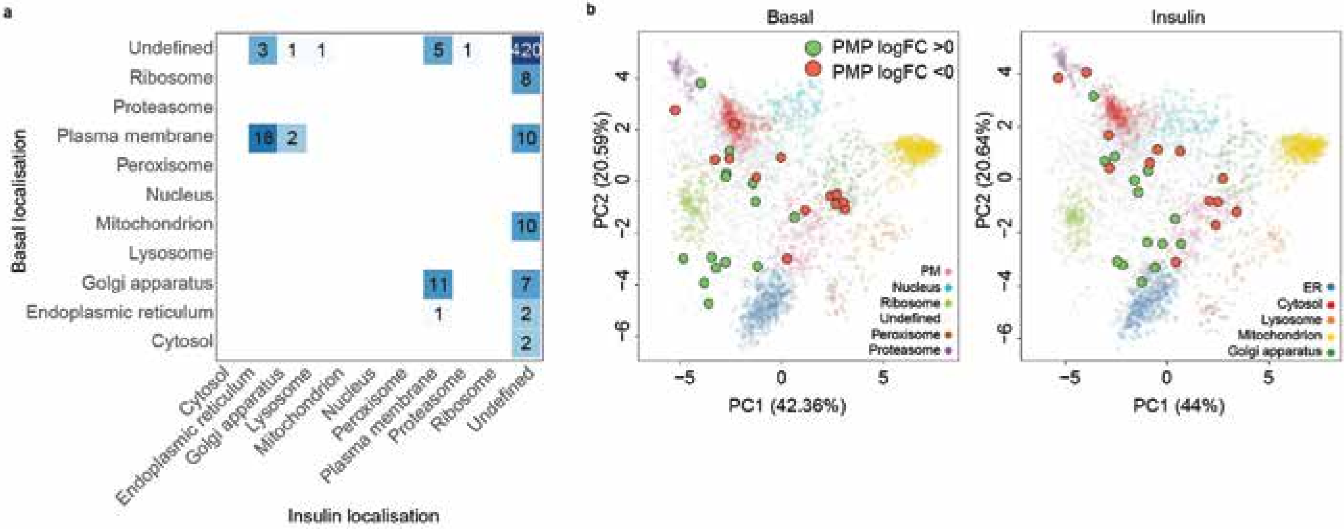

Supplementary Figure 3: a) Organelle assignment of proteins predicted to relocalise with high-confidence (*diff. loc. prob*. = 1) under basal and insulin-treated conditions. b) PCA projection of LOPIT-DC adipocyte subcellular map under basal (left) and insulin-stimulated (right) conditions with proteins predicted to move with high-confidence (*diff. loc. prob*. = 1) also up (green) or downregulated (red) ($p<0.05$) in plasma membrane proteomics (PMP) data highlighted. Organelle marker proteins coloured as red = cytosol, dark blue = endoplasmic reticulum, dark green = Golgi apparatus, orange = lysosome, yellow = mitochondrion, light blue = nucleus, brown = peroxisome, pink = plasma membrane, purple = proteasome, green = ribosome, grey = undefined. Source data are provided as a Source Data File.

Supplementary Figure 4

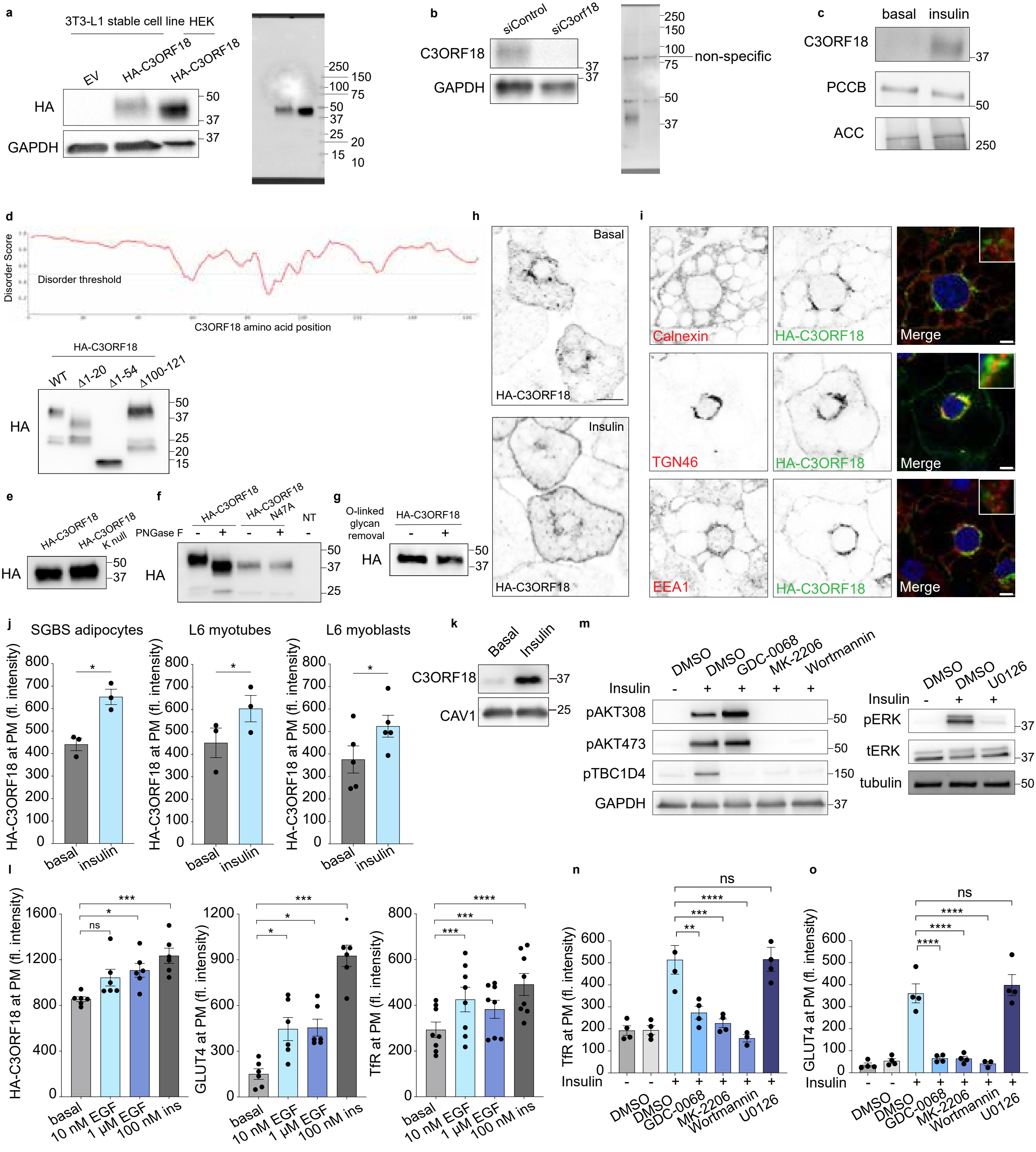

Supplementary Figure 4: a) Western blot analysis of HA-C3ORF18 expression in 3T3-L1 adipocytes stable cell lines expressing HA-C3ORF18 or the empty control vector (EV), and HEK-293 cells transiently overexpressing HA-C3ORF18. 4-20% SDS-PAGE gel used to resolve proteins, full membrane shown on right. b) Western blot analysis of C3ORF18 expression in 3T3-L1 adipocytes with siRNA-mediated depletion of C3ORF18. Knockdown was performed on day 6 and day 10 of differentiation and cells were harvested on day 14 (8 d knockdown). 10% SDS-PAGE gel used to resolve proteins, full membrane shown on right. c) Western blot analysis of C3ORF18 in the affinity-purified PM fraction of differentiated (day 10) 3T3-L1 adipocytes isolated for plasma membrane proteomics as described in Fig. 3b. d-g) Western blot analysis of HA-C3ORF18 variants transiently overexpressed in HEK-293 cells to determine the cause of the apparent molecular weight shift. d) Intrinsic disorder prediction of C3ORF18 (upper panel) generated using the IUPred3A server (iupred.elte.hu)[92]. Y-axis represents the disorder probability score, and x-axis shows amino acid position. The grey horizontal line (0.5) indicates the disorder threshold, with regions above this value predicted to be intrinsically disordered. HA-C3ORF18 with indicated amino acid residues deleted (lower panel) revealed that the disordered N-terminus is responsible for most of the apparent molecular weight increase observed by SDS-PAGE. e) Deletion of all lysine (K) residues did not alter the apparent molecular weight of C3ORF18, ruling out lysine modifications as the cause of the molecular weight shift. f) PNGase treatment, and mutation of Asn47 to Ala (N47A) increased C3ORF18 migration by SDS-PAGE, indicative of N-linked glycosylation at Asn47. The shift in the apparent molecular weight after N-glycan removal suggests that N-linked glycosylation contributes, albeit marginally, to the higher-than-expected observed molecular weight for C3ORF18. g) O-glycan removal did not alter the apparent molecular weight of C3ORF18. h) Immunostaining of HA-C3ORF18 overexpressing 3T3-L1 adipocytes under basal and insulin-stimulated (100 nM, 30 min) conditions (scale bar = 10 µm). i) Representative images of calnexin, TGN46 and EEA1 immunostaining in HA-C3ORF18 overexpressing 3T3-L1 adipocytes (scale bar = 10 µm). j) Relative fluorescence intensity of anti-HA surface staining in basal and insulin-stimulated HA-C3ORF18 expressing SGBS adipocytes, L6 myoblasts and myotubes (n = 3-6 biological replicates). k) Western blot analysis of C3ORF18 abundance in plasma membrane from primary rat adipocytes stimulated with 20 nM insulin for 20 min (Caveolin 1 (Cav1) as loading control, n = 3 biological replicates). l) Relative fluorescence intensity of anti-HA, GLUT4 and TfR surface staining in 3T3-L1 adipocytes following stimulation with 10 nM or 1 µM EGF or 100 nM insulin for 5 min. m) Western blot analysis of 3T3-L1 adipocytes treated with AKT-PI3K (GDC-0068, MK-2206, Wortmannin) or MAPK signalling inhibitors (U0126) for 15 min prior to insulin stimulation (100 nM, 30 min). Representative blot shown, n = 3 biological replicates. n-o) Relative fluorescence intensity of surface TfR (n) and GLUT4 (o) in 3T3-L1 adipocytes treated with AKT-PI3K (GDC-0068, MK-2206, Wortmannin) or MAPK signalling inhibitors (U0126) prior to insulin stimulation (100 nM, 30 min; n = 4 biological replicates for 0 nM insulin, DMSO, GDC-068, MK-2206, U0126; n = 3 for biological replicates for Wortmannin). All data represented as mean ± SEM (j, k, m, n). n.s. non-significant; *$p < 0.05$; **$p < 0.01$; ***$p < 0.01$; ****$p < 0.0001$ by paired two-tailed Student's *t*-test (j) or one-way ANOVA with Šidák's multiple comparisons test (k, n, o). Source data are provided as a Source Data File.

Supplementary Figure 5

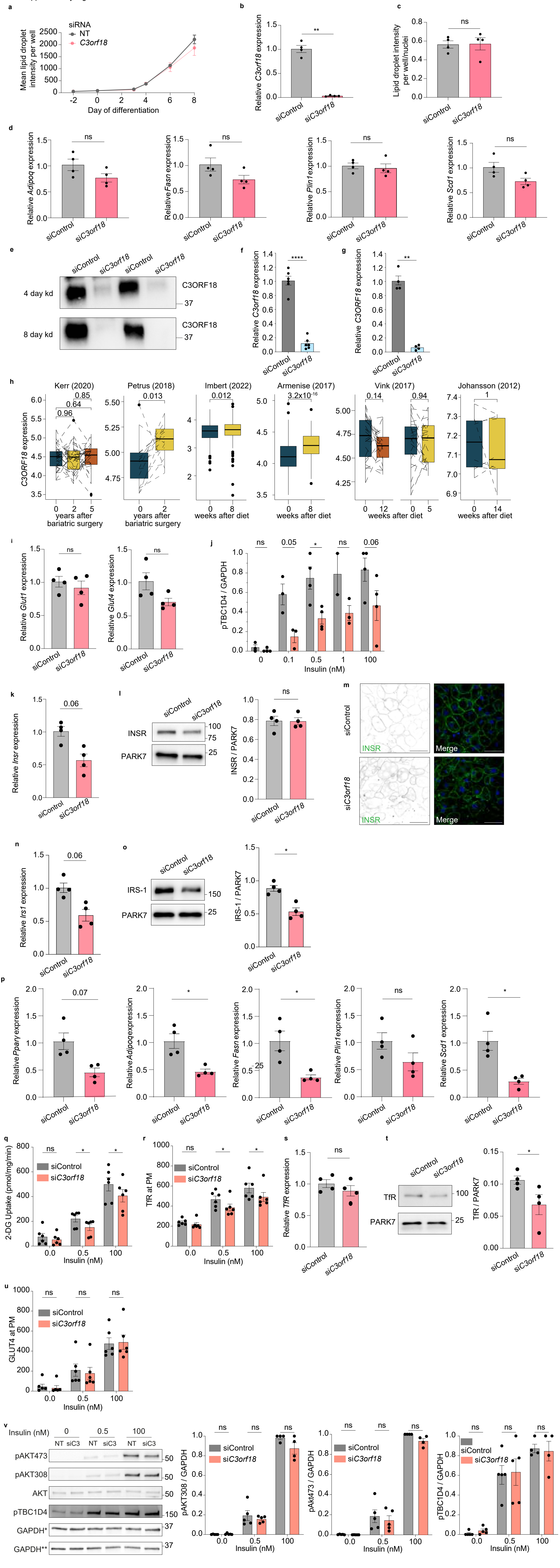

Supplementary Figure 5: a) Longitudinal analysis of mean lipid droplet intensity per well in differentiating 3T3-L1 adipocytes by digital phase contrast imaging (n = 4 biological replicates). b) Relative mRNA expression of *C3orf18* in control (siControl) and *C3orf18* knockdown (si*C3orf18*) 3T3-L1 cells on day 8 of adipogenesis (n = 4 biological replicates). c) Average lipid droplet intensity normalised to total nuclei number for each sample at day 8 of adipogenesis. d) Relative mRNA expression of indicated gene in control (siControl) and *C3orf18* knockdown (si*C3orf18*) 3T3-L1 cells on day 8 of adipogenesis (n = 4 biological replicates). e) Western blot of C3ORF18 expression in 3T3-L1 adipocytes following 4 or 8 d knockdown post-differentiation. f-g) Relative expression of *C3ORF18* in (f) 3T3-L1 and (g) SGBS adipocytes after 8 d knockdown post-differentiation (normalised to β-actin, n = 4 (f) and 6 (g) biological replicates). h) *C3ORF18* mRNA expression in adipose tissue following weight loss. Figure generated from adiposetissue.org[26] using data from[27-47]. i) Relative mRNA expression (normalised to β-actin) of *GLUT1 (Slc2a1)* and *GLUT4 (Slc2a4)* in 3T3-L1 adipocytes following 8 d *C3orf18* knockdown post-differentiation (n = 4 biological replicates). j) Quantification of pT642 TBC1D4 in 3T3-L1 adipocytes following 8 d *C3orf18* depletion from differentiated adipocytes, stimulated with 0, 0.1, 0.5, 1 or 100 nM insulin for 30 min. Normalised to GAPDH (n = 4 biological replicates for 0 nM si*C3orf18*, 0.5 and 100 nM; n = 3 for 0 nM siControl, 0.1 nM and 1 nM si*C3orf18*; n = 2 for 1 nM siControl; representative blot shown in Fig. 5g; pale red = *C3orf18* siRNA, grey = non-targeting control siRNA). k) Relative mRNA expression of *Insr* following 8 d *C3orf18* knockdown post-differentiation (normalised to β-actin). l) Western blot and quantification of INSR protein expression in 3T3-L1 adipocytes following 8 d *C3orf18* knockdown post-differentiation (n = 4 biological replicates). m) Immunostaining of INSR in 3T3-L1 adipocytes under basal condition following 8 d *C3orf18* knockdown post-differentiation (scale bar = 50 μm). n) Relative mRNA expression (normalised to β-actin) and (o) Western blot and quantification of IRS-1 protein expression in 3T3-L1 adipocytes following 8 d *C3orf18* knockdown post-differentiation (n = 4 biological replicates). p) Relative mRNA expression of indicated genes in 3T3-L1 adipocytes after 8 d *C3orf18* knockdown (n = 4 biological replicates). q) 2-deoxyglucose (2-DG) uptake in 3T3-L1 adipocytes following 4 d *C3orf18* knockdown post-differentiation (n = 6 biological replicates; pale red = *C3orf18* siRNA, grey = non-targeting control siRNA). r) Fluorescence intensity of surface TfR in 3T3-L1 adipocytes stimulated with 0.5 or 100 nM insulin for 30 min following 4 d *C3orf18* knockdown post-differentiation (n = 6 biological replicates). s) Relative mRNA expression of *TfR* following 4 d *C3orf18* knockdown post-differentiation (normalised to β-actin). t) Western blot and quantification of TfR protein expression in 3T3-L1 adipocytes following 4 d *C3orf18* knockdown post-differentiation (n = 4 biological replicates). u) Fluorescence intensity of surface GLUT4 in 3T3-L1 adipocytes stimulated with 0.5 or 100 nM insulin for 30 min following 4 d *C3orf18* knockdown post-differentiation (n = 6 biological replicates). v) Representative Western blot and quantification of phosphorylated AKT (Thr308 and Ser473) and TBC1D4 (Thr642) in 3T3-L1 adipocytes following 4 d *C3orf18* depletion post-differentiation, stimulated with 0, 0.1, 0.5, 1 or 100 nM insulin for 30 min (n = 5 biological replicates for 0 and 0.5 nM; n = 4 for 100 nM). GAPDH* loading control for pAKT308 and pTBC1D4, GAPDH** loading control for pAKT473 and total AKT, NT is non-targeting siControl. Data in (h) analysed using Wilcoxon's matched pairs test. All data represented as mean ± SEM (b-d, f, g, i-l, n-v). n.s. non-significant; *$p < 0.05$; **$p < 0.01$; ****$p < 0.0001$ by paired two-tailed Student's *t*-test (b-d, f, g, i, k, l, n-p, s, t), two-way ANOVA with Sidak's multiple comparisons test (q, r, u) or by a mixed effects model with Šidák's multiple comparisons test (j,v). Source data are provided as a Source Data File.

References (only cited in Supplemental Information)

92. Erdős G & Dosztányi Z. AIUPred: combining energy estimation with deep learning for the enhanced prediction of protein disorder. *Nucleic Acis Res* 52(W1):W176-W181 (2024).
