# Supplementary material for: Dynamic subcellular proteomics identifies regulators of adipocyte insulin action
Source: Nat Commun. 2026 Feb 28;17:3310. doi: 10.1038/s41467-026-70116-9 (PMC13066455; doi:10.1038/s41467-026-70116-9)
Supplement: Supplementary file 2 — Description of Additional Supplementary Files [file 41467_2026_70116_MOESM2_ESM.pdf]

Description of Additional Supplementary Files

File Name: Supplementary Data 1

Description: Excel file for data relating to LOPIT-DC data, containing details on centrifugation speeds used in LOPIT-DC to fractionate adipocytes (sheet 2), TMT labelling (sheet 3), proteins used as marker proteins for each organelle as training data for BANDLE (sheet 3), protein organelle allocations by BANDLE (sheet 4), comparison of protein localisation in 3T3-L1 vs. SGBS adipocytes (Klingelhuber et al., 2024) (sheet 5), Gene ontology cellular compartment analysis for proteins assigned to each organelle (not including designated marker proteins), top 5 terms with p.adj <0.05 (Benjamini-Hochberg adjusted) shown (sheet 6), insulin-regulated phosphosites in proteins identified in LOPIT-DC in Fazakerley (2023)[10] and Humphrey (2013)[1] (sheet 7), insulin-regulated phophosites of selected proteins in Fazakerley (2023)[10] (sheet 8), insulin-regulated phosphosites of selected proteins in Humphrey (2013)[1] (fold change -/+ 2 at any timepoint in insulin time course (0-60 min) (sheet 9), Gene ontology biological processes, REACTOME and KEGG pathway enrichment analysis of proteins with differential localisation probability = 1 in LOPIT-DC, pathways with p.adj <0.05 (Benjamini-Hochberg adjusted) shown (sheet 10).

File Name: Supplementary Data 2

Description: Excel file for data relating to plasma membrane profiling, containing details of TMT labelling (sheet 2), fold change and P values (adjusted for multiple comparisons using the Benjamini-Hochberg method) of proteins at the plasma membrane following insulin stimulation (sheet 3), and list of proteins with differential localisation probability = 1 in LOPIT-DC and fold change (insulin/basal) = adj.p <0.05 (Benjamini-Hochberg adjusted) in PM proteomics data.

File Name: Supplementary Data 3

Description: Excel file containing qpCR primers and Taqman Probe IDs (sheet 1), antibodies used for Western blotting (sheet 2) and immunofluorescence microscopy (sheet 3), and plasmids used in this study (sheet 4).
