# Supplementary material for: Dynamic subcellular proteomics identifies regulators of adipocyte insulin action
Source: Nat Commun. 2026 Feb 28;17:3310. doi: 10.1038/s41467-026-70116-9 (PMC13066455; doi:10.1038/s41467-026-70116-9)
Supplement: Supplementary file 6 — Reporting Summary [file 41467_2026_70116_MOESM6_ESM.pdf]

nature portfolio

Corresponding author(s): Daniel Fazakerley

Last updated by author(s): 29/04/2025

Reporting Summary

Nature Portfolio wishes to improve the reproducibility of the work that we publish. This form provides structure for consistency and transparency in reporting. For further information on Nature Portfolio policies, see our Editorial Policies and the Editorial Policy Checklist.

Statistics

For all statistical analyses, confirm that the following items are present in the figure legend, table legend, main text, or Methods section.

n/a	Confirmed	
☐	☒	The exact sample size (*n*) for each experimental group/condition, given as a discrete number and unit of measurement
☐	☒	A statement on whether measurements were taken from distinct samples or whether the same sample was measured repeatedly
☐	☒	The statistical test(s) used AND whether they are one- or two-sided<br>*Only common tests should be described solely by name; describe more complex techniques in the Methods section.*
☒	☐	A description of all covariates tested
☐	☒	A description of any assumptions or corrections, such as tests of normality and adjustment for multiple comparisons
☐	☒	A full description of the statistical parameters including central tendency (e.g. means) or other basic estimates (e.g. regression coefficient) AND variation (e.g. standard deviation) or associated estimates of uncertainty (e.g. confidence intervals)
☐	☒	For null hypothesis testing, the test statistic (e.g. *F*, *t*, *r*) with confidence intervals, effect sizes, degrees of freedom and *P* value noted<br>*Give P values as exact values whenever suitable.*
☐	☒	For Bayesian analysis, information on the choice of priors and Markov chain Monte Carlo settings
☒	☐	For hierarchical and complex designs, identification of the appropriate level for tests and full reporting of outcomes
☒	☐	Estimates of effect sizes (e.g. Cohen's *d*, Pearson's *r*), indicating how they were calculated

Our web collection on statistics for biologists contains articles on many of the points above.

Software and code

Policy information about availability of computer code

Data collection

LOPIT: MS analysis was performed using an Lumos Orbitrap mass spectrometer coupled to a Dionex Ultimate 3000 RSLC nanoUPLC system (Thermo Fisher Scientific, Waltham, MA, USA).
Plasma Membrane Proteomics: TMT-labelled samples were analysed using an Orbitrap Fusion Lumos (Thermo Fisher Scientific).
Protein assay data was collected using a Tecan Spark 10M Plate Reader.
Western blot analysis was carried out using the Chemidoc MP (Bio-Rad).
2-DG uptake was quantified on the TriCarb 2900TR (PerkinElmer).
RT-PCR was performed on the ABI QuantStudio 5.
Confocal imaging data were acquired using the Opera Phenix (Perkin Elmer) and Leica SP8 microscope.

Data analysis

LOPIT: Raw LC-MS data files were processed with Proteome Discoverer v2.5 (Thermo Fisher Scientific) using the Mascot server 2.8.3 (Matrix Science). LOPIT analysis was performed in All proteomics data analysis was performed in R (v4.3.2) using the Bioconductor packages pRoloc (v1.42.0), bandle (v1.6), clusterProfiler (v4.10.1) and ggplot2 (v3.5.1) and all code is provided here: https://github.com/CambridgeCentreForProteomics/adipocyteLOPIT2025

Plasma Membrane Proteomics: Mass spectra were processed using a Sequest-based software pipeline for quantitative proteomics, 'MassPike', through a collaborative arrangement with Professor Steven Gygi's laboratory at Harvard Medical School. Analysis was performed in Perseus version 1.5.2.20.4.

Data handling was performed in Microsoft Excel.

Statistical analsysis was performed using GraphPad Prism 10.
Western blot images were quantified using ImageLab (v6.1, Bio-Rad).
Microscopy images were analysed using Harmony High-Content Imaging and Analysis Software or ImageJ Fiji v2.9.0.

For manuscripts utilizing custom algorithms or software that are central to the research but not yet described in published literature, software must be made available to editors and reviewers. We strongly encourage code deposition in a community repository (e.g. GitHub). See the Nature Portfolio guidelines for submitting code & software for further information.

Data

Policy information about availability of data

All manuscripts must include a data availability statement. This statement should provide the following information, where applicable:

- Accession codes, unique identifiers, or web links for publicly available datasets
- A description of any restrictions on data availability
- For clinical datasets or third party data, please ensure that the statement adheres to our policy

Proteomic raw data are available via PRIDE with the identifiers PXD061017 (LOPIT-DC 819 data) and PXD061616 (plasma membrane data). These data will be fully accessible upon manuscript acceptance.

Project Name: Dynamic subcellular proteomics identifies novel regulators of adipocyte insulin action
Project accession: PXD061017
Project DOI: Not applicable
Reviewer access details: Log in to the PRIDE website using the following details: Project accession: PXD061017 Token: ViogHCm8xHUI
Alternatively, reviewer can access the dataset by logging in to the PRIDE website using the following account details: Username: reviewer_pxd061017@ebi.ac.uk
Password: Vn0tGXdlBmrB

Project Name: Dynamic subcellular proteomics identifies novel regulators of adipocyte insulin action
Project accession: PXD061616
Project DOI: Not applicable
Reviewer access details: Log in to the PRIDE website using the following details: Project accession: PXD061616 Token: HI27FSmoEpEx
Alternatively, reviewer can access the dataset by logging in to the PRIDE website using the following account details: Username: reviewer_pxd061616@ebi.ac.uk
Password: AOP5b8s2cXgs

Research involving human participants, their data, or biological material

Policy information about studies with human participants or human data. See also policy information about sex, gender (identity/presentation), and sexual orientation and race, ethnicity and racism.

Reporting on sex and gender	N/A
Reporting on race, ethnicity, or other socially relevant groupings	N/A
Population characteristics	N/A
Recruitment	N/A
Ethics oversight	N/A

Note that full information on the approval of the study protocol must also be provided in the manuscript.

Field-specific reporting

Please select the one below that is the best fit for your research. If you are not sure, read the appropriate sections before making your selection.

[x] Life sciences [ ] Behavioural & social sciences [ ] Ecological, evolutionary & environmental sciences

For a reference copy of the document with all sections, see nature.com/documents/nr-reporting-summary-flat.pdf

Life sciences study design

All studies must disclose on these points even when the disclosure is negative.

Sample size	For LOPIT, n=3 for basal and n=3 insulin samples.<br>For plasma membrane proteomics, n=6 for basal and n=6 for insulin samples.<br>We typically performed n=3-6 biological replicates per experiment, informed by pilot experiments. Individual data points (per biological replicate) were plotted where possible.
Data exclusions	We have not excluded any data from analysis.

Replication	All attempts at replication performed were successful. Further, where possible, we used orthoganal methods to validate observations (e.g., immunofluorescence to validate changes in plasma membrane C3ORF18 abundance)
Randomization	Cells were randomly assigned to experimental conditions. The placement of specifc treatments within culture plates was randomised. For knockdown experiments, cells were pooled before incubation with siRNA so there was no difference between cells prior to knockdown.
Blinding	In general, investigators were not blinded to experimental groups since treatments and experiments were perfrmed by the same investigator. The exception is the 2DOG uptake assays - the investigator performing the 2DOG uptake assay was blinded to experimental groups.

Reporting for specific materials, systems and methods

We require information from authors about some types of materials, experimental systems and methods used in many studies. Here, indicate whether each material, system or method listed is relevant to your study. If you are not sure if a list item applies to your research, read the appropriate section before selecting a response.

Materials & experimental systems

n/a	Involved in the study
☐	☒ Antibodies
☐	☒ Eukaryotic cell lines
☒	☐ Palaeontology and archaeology
☐	☒ Animals and other organisms
☒	☐ Clinical data
☒	☐ Dual use research of concern
☒	☐ Plants

Methods

n/a	Involved in the study
☒	☐ ChIP-seq
☒	☐ Flow cytometry
☒	☐ MRI-based neuroimaging

Antibodies

Antibodies used

The following antibodies were used for western blotting:
C3ORF18 Novus NBP1-59970
C3ORF18 Novus biologicals NBP1-83405
FASN Cell Signaling Technology 3180
PLIN1 Cell Signaling Technology 9349
Calnexin Abcam ab22595
HA BioLegend 901515
GAPDH Cell Signaling Technology 2118
PCCB Invitrogen PA5-21647
ACC Cell Signaling Technology 3676
pAkt308 Cell Signaling Technology 13038
pAkt473 Cell Signaling Technology 4060
pAS160 Cell Signaling Technology 4288
pERK1/3 Cell Signaling Technology 9101
ERK1/2 Cell Signaling Technology 9102
alpha tubulin Sigma-Aldrich T9026
panAkt Cell Signaling Technology 2920
INSR Cell Signaling Technology 23413S
GLUT1 Cell Signaling Technology 12939
GLUT4 Gift from Prof. David James, University of Sydney
TFR Invitrogen 136800
CAVEOLIN Cell Signaling Technology 3267T
IRS1 Cell Signaling Technology 3407S
O-GlcNAc Cell Signaling Technology 82332
PARK7 Abcam ab76008
PARK7 Abcam ab283314
Goat anti-Rabbit IgG, HRP Invitrogen G-21234
Goat anti-Mouse IgG, HRP Invitrogen 31430
Goat anti-Rabbit Alexa Fluor 488 Invitrogen A32731

The following antibodies were used for immunofluorescence:
C3ORF18 Invitrogen PA5-113528
HA BioLegend 901515
TfR Invitrogen 14-0711
GLUT4 (surface staining) Integral Molecular LM048
GLUT4 (colocalisation) kind gift from Geoff Holman and Francoise Koumanov
Septin 2 Abcam AB187654

Septin 11 Proteintech 14672-1-AP
PLIN1 Progen GP29
EEA1 Abcam ab109110
TGN46 Abcam ab16059
Calnexin Abcam ab22595
C3ORF18 Novus biologicals NBP1-83405
INSR Cell Signaling Technology 23413S
Goat anti-Mouse IgG Alexa Fluor 488 Invitrogen A11001
Goat anti-Human IgG Alexa Fluor 647 Invitrogen A48279
Goat anti-Rat IgG Alexa Fluor 568 Invitrogen A11077
Goat anti-Rabbit Alexa Fluor 488 Invitrogen A32731
Goat anti-Rabbtit Alexa Fluor 647 Invitrogen A32733

Validation

All the available information on antibody validation can be found in the following manufacturer links or publications provided below. Manufacturer links contain details of validation and provide citations of studies that have used these antibodies in similar applications.

C3ORF18 Novus NBP1-59970 (validated using siRNA in this paper): https://www.novusbio.com/products/c3orf18-antibody_nbp1-59970?srsltid=AfmBOoppw7dQNBEMhzrh6MB1Myh4isxJ2LQ7ZY8CHgxmCyS8QyBDM1ry
C3ORF18 Novus biologicals NBP1-83405 (validated using siRNA in this paper): https://www.novusbio.com/products/c3orf18-antibody_nbp1-83405
FASN Cell Signaling Technology 3180: https://www.cellsignal.com/products/primary-antibodies/fatty-acid-synthase-c20g5-rabbit-mab/3180?srsltid=AfmBOoquM4SorbRpSXIlUYPPAeINNNgWAUvxfn5DrEvZacxDX2g_yGde
PLIN1 Cell Signaling Technology 9349: https://www.cellsignal.com/products/primary-antibodies/perilipin-1-d1d8-xp-rabbit-mab/9349?srsltid=AfmBOoqGTOfZSNwnTH07ZiqmdFsNmyo6N13xSe-e6vu1yNRcrP8LoNUG
Calnexin Abcam ab22595: https://www.abcam.com/en-us/products/primary-antibodies/calnexin-antibody-er-marker-ab22595?srsltid=AfmBOoq-QyyNavxNmFOVKfwd80KoqJAnenZaT_fl8gC12AFdxfF4V6Tw
HA BioLegend 901515: https://www.biolegend.com/en-gb/products/anti-ha-11-epitope-tag-antibody-11071
GAPDH Cell Signaling Technology 2118: https://www.cellsignal.com/products/primary-antibodies/gapdh-14c10-rabbit-mab/2118?srsltid=AfmBOorv6aeMDmlJDM3Tvqp2LH1mYZsfdqcXbvj2JtOEb-M8zgjJu8vw
PCCB Invitrogen PA5-21647: https://www.thermofisher.com/antibody/product/PCCB-Antibody-Polyclonal/PA5-21647
ACC Cell Signaling Technology 3676: https://www.cellsignal.com/products/primary-antibodies/acetyl-coa-carboxylase-c83b10-rabbit-mab/3676?srsltid=AfmBOooaWjmmyEFaE58ouO00ztVg8bsF172Tvyfu8nM6eWls0ru8rYIm
pAkt308 Cell Signaling Technology 13038: https://www.cellsignal.com/products/primary-antibodies/phospho-akt-thr308-d25e6-xp-rabbit-mab/13038?srsltid=AfmBOordDSwYDXPmwk7qVCFSbNna9tC7XQq9XV65uagfuiDZ48CDZ18e
pAkt473 Cell Signaling Technology 4060: https://www.cellsignal.com/products/primary-antibodies/phospho-akt-ser473-d9e-xp-rabbit-mab/4060?srsltid=AfmBOopOeEWedepWy7EgKXQ31rfGEoylOIN5oND0KxZhiQVg6QHGBEsl
pAS160 Cell Signaling Technology 4288: https://www.cellsignal.com/products/primary-antibodies/phospho-as160-thr642-antibody/4288?srsltid=AfmBOorNtZR0sQ63XxOPHQKG4WE2_P4le8A6vwhAbQbgy5jLYOwvGICt
pERK1/3 Cell Signaling Technology 9101: https://www.cellsignal.com/products/primary-antibodies/phospho-p44-42-mapk-erk1-2-thr202-tyr204-antibody/9101?srsltid=AfmBOorF-gUdAoEkgX5BcDCDSAjBz8aQRBd7ZgZUCr2KxiWjWimsNXFl
ERK1/2 Cell Signaling Technology 9102: https://www.cellsignal.com/products/primary-antibodies/p44-42-mapk-erk1-2-antibody/9102?srsltid=AfmBOopClt8PnivBir30HmP4AMDRmgpgbb6RVyO3UWXLLmu-CQi8sB14
alpha tubulin Sigma-Aldrich T9026: https://www.sigmaaldrich.com/GB/en/product/sigma/t9026
panAkt Cell Signaling Technology 2920: https://www.cellsignal.com/products/primary-antibodies/akt-pan-40d4-mouse-mab/2920?srsltid=AfmBOoq5W-orCHETAYyGcphmon1gg9aCqV3N2RayHWRc5Xfdhhyjxv2j
INSR Cell Signaling Technology 23413S: https://www.cellsignal.com/products/primary-antibodies/insulin-receptor-beta-e9l5v-rabbit-monoclonal-antibody/23413?srsltid=AfmBOoq-S7Yd2_bAmVxCQEAPWrSjCEgM3StuhuaQ6J8dTTZffCRKB_Yl
GLUT1 Cell Signaling Technology 12939
rabbit anti-GLUT4 antibody (Courtesy of Professor David James, University of Sydney). Antibody specificity was confirmed by comparing samples with Slc2a4 siRNA knockdown and those with non-targeting control through western blot and immunofloresence imaging to assess the binding of this Ab to GLUT4 protein - PMID: 37291194
TFR Invitrogen 136800: https://www.thermofisher.com/antibody/product/Transferrin-Receptor-Antibody-clone-H68-4-Monoclonal/13-6800
CAVEOLIN Cell Signaling Technology 3267T: https://www.cellsignal.com/products/primary-antibodies/caveolin-1-d46g3-rabbit-monoclonal-antibody/3267?srsltid=AfmBOoqTjGBiB2_eofk1Mfr77Eb1u98pkt0cO5KGszJUASTQU1VPi4lQ
IRS1 Cell Signaling Technology 3407S: https://www.cellsignal.com/products/primary-antibodies/irs-1-d23g12-rabbit-monoclonal-antibody/3407
O-GlcNAc Cell Signaling Technology 82332: https://www.cellsignal.com/products/primary-antibodies/o-glcnac-multimab-rabbit-monoclonal-antibody-mix/82332
PARK7 Abcam ab76008: https://www.abcam.com/en-us/products/primary-antibodies/park7-dj1-antibody-ep2815y-ab76008
PARK7 Abcam ab283314: https://www.abcam.com/en-us/products/primary-antibodies/park7-dj1-antibody-ep2815y-mouse-igg1-chimeric-ab283314

Goat anti-Rabbit IgG, HRP Invitrogen G-21234: https://www.thermofisher.com/antibody/product/Goat-anti-Rabbit-IgG-H-L-Cross-Adsorbed-Secondary-Antibody-Polyclonal/G-21234
Goat anti-Mouse IgG, HRP Invitrogen 31430: https://www.thermofisher.com/antibody/product/Goat-anti-Mouse-IgG-H-L-Secondary-Antibody-Polyclonal/31430
Goat anti-Rabbit Alexa Fluor 488 Invitrogen A32731: https://www.thermofisher.com/antibody/product/Goat-anti-Rabbit-IgG-H-L-Highly-Cross-Adsorbed-Secondary-Antibody-Polyclonal/A32731

The following antibodies were used for immunofluorescence:
C3ORF18 Invitrogen PA5-113528: https://www.thermofisher.com/antibody/product/C3orf18-Antibody-Polyclonal/PA5-113528
HA BioLegend 901515: https://www.biolegend.com/en-gb/products/anti-ha-11-epitope-tag-antibody-11071
TfR Invitrogen 14-0711 : https://www.thermofisher.com/antibody/product/CD71-Transferrin-Receptor-Antibody-clone-R17217-RI7-217-1-4-Monoclonal/14-0711-82
GLUT4 (surface staining) Integral Molecular, kindly provided by Joseph Tucker, Integral Molecular, PA, USA): Tucker et al. (2018); PNAS https://www.pnas.org/doi/10.1073/pnas.1716788115
GLUT4 (colocalisation) kind gift from Geoff Holman and Francoise Koumanov. Affinity purified in house and validated using siRNA knockdown of GLUT4.
Septin 2 Abcam AB187654: https://www.abcam.com/en-us/products/primary-antibodies/septin-2-antibody-epr12122-ab187654?srsltid=AfmBOop_39pSQ3FppZgyu79mluXJzBfPO0R9Nqfi9z3nrsHVpD-V5pnG
Septin 11 Proteintech 14672-1-AP: https://www.ptglab.com/products/SEPT11-Antibody-14672-1-AP.htm?srsltid=AfmBOorBwmiWUjWUb9p0vWXRRvbjuhi984J_3xcZ2iVWa4JNK9sMGZ3e
PLIN1 Progen GP29: https://www.progen.com/anti-Perilipin-1-N-terminus-guinea-pig-polyclonal-serum/GP29
EEA1 Abcam ab109110: https://www.abcam.com/en-us/products/primary-antibodies/eea1-antibody-epr4245-early-endosome-marker-ab109110
TGN46 Abcam ab16059:https://www.abcam.com/en-us/products/primary-antibodies/tgn46-antibody-golgi-marker-ab16059
Calnexin Abcam ab22595: https://www.abcam.com/en-us/products/primary-antibodies/calnexin-antibody-er-marker-ab22595
C3ORF18 Novus biologicals NBP1-83405 (validated using siRNA in this paper): https://www.novusbio.com/products/c3orf18-antibody_nbp1-83405
INSR Cell Signaling Technology 23413S: https://www.cellsignal.com/products/primary-antibodies/insulin-receptor-beta-e9l5v-rabbit-monoclonal-antibody/23413?srsltid=AfmBOoq-S7Yd2_bAmVxCQEAPWrSjCEgM3StuhuaQ6J8dTTZffCRKB_Yl

Goat anti-Mouse IgG Alexa Fluor 488 Invitrogen A11001: https://www.thermofisher.com/antibody/product/Goat-anti-Mouse-IgG-H-L-Cross-Adsorbed-Secondary-Antibody-Polyclonal/A-11001
Goat anti-Human IgG Alexa Fluor 647 Invitrogen A48279: https://www.thermofisher.com/antibody/product/Goat-anti-Human-IgG-H-L-Cross-Adsorbed-Secondary-Antibody-Polyclonal/A48279
Goat anti-Rat IgG Alexa Fluor 568 Invitrogen A11077: https://www.thermofisher.com/antibody/product/Goat-anti-Rat-IgG-H-L-Cross-Adsorbed-Secondary-Antibody-Polyclonal/A-11077
Goat anti-Rabbit Alexa Fluor 488 Invitrogen A32731: https://www.thermofisher.com/antibody/product/Goat-anti-Rabbit-IgG-H-L-Highly-Cross-Adsorbed-Secondary-Antibody-Polyclonal/A32731
Goat anti-Rabbtit Alexa Fluor 647 Invitrogen A32733: https://www.thermofisher.com/antibody/product/Goat-anti-Rabbit-IgG-H-L-Highly-Cross-Adsorbed-Secondary-Antibody-Polyclonal/A32733

Eukaryotic cell lines

Policy information about cell lines and Sex and Gender in Research

Cell line source(s)	Mouse 3T3-L1 fibroblasts were kindly provided by David James (University of Sydney, Australia), and were originally from Howard Green (Harvard Medical School, Boston, MA) (RRID: CVCL_0A20). Rat L6 myoblasts were kindly provided by David James (University of Sydney, Australia), and were originally from David Yaffe (Weizmann Institute of Science, Israel) (RRID: CVCL_0385). HEK cells were from David James (University of Sydney, Australia). SGBS adipocytes were provided by Professor Martin Wabitsch (Division of Pediatric Endocrinology and Diabetes, University Medical Center Ulm, Germany).
Authentication	3T3-L1 and SGBS cell lines were not authenticated, but they were differentiated into adipocytes and only used if they reached >90-95% differentiation as observed by lipid accumulation. L6 cell line was not authenticated but were only used post-differentiation, as confirmed by their morphology and formation of myotubes under known differentiation conditions. HEK cells were not authenticated.
Mycoplasma contamination	3T3-L1, L6 and SGBS cells tested negative for mycoplasma. HEK cells were not tested for mycoplasma.
Commonly misidentified lines (See ICLAC register)	None used.

Animals and other research organisms

Policy information about studies involving animals; ARRIVE guidelines recommended for reporting animal research, and Sex and Gender in Research

Laboratory animals	Male Wistar Rats
Wild animals	*Provide details on animals observed in or captured in the field; report species and age where possible. Describe how animals were caught and transported and what happened to captive animals after the study (if killed, explain why and describe method; if released, say where and when) OR state that the study did not involve wild animals.*
Reporting on sex	For the purpose of preparing primary adipocytes from epididymal fat pads, only male rats could be used.
Field-collected samples	*For laboratory work with field-collected samples, describe all relevant parameters such as housing, maintenance, temperature, photoperiod and end-of-experiment protocol OR state that the study did not involve samples collected from the field.*

Ethics oversight	Ethical approval was granted by the University of Bath Animal Welfare and Ethical Review Body, and all animal procedures were conducted in accordance with UK Home Office regulations.

Note that full information on the approval of the study protocol must also be provided in the manuscript.

Plants

Seed stocks	*Report on the source of all seed stocks or other plant material used. If applicable, state the seed stock centre and catalogue number. If plant specimens were collected from the field, describe the collection location, date and sampling procedures.*
Novel plant genotypes	*Describe the methods by which all novel plant genotypes were produced. This includes those generated by transgenic approaches, gene editing, chemical/radiation-based mutagenesis and hybridization. For transgenic lines, describe the transformation method, the number of independent lines analyzed and the generation upon which experiments were performed. For gene-edited lines, describe the editor used, the endogenous sequence targeted for editing, the targeting guide RNA sequence (if applicable) and how the editor was applied.*
Authentication	*Describe any authentication procedures for each seed stock used or novel genotype generated. Describe any experiments used to assess the effect of a mutation and, where applicable, how potential secondary effects (e.g. second site T-DNA insertions, mosiacism, off-target gene editing) were examined.*
